# Associations between maternal vitamin D status and porcine litter characteristics throughout gestation

**DOI:** 10.1186/s40104-022-00760-w

**Published:** 2022-09-20

**Authors:** Claire Stenhouse, Emma Hurst, Richard J. Mellanby, Cheryl J. Ashworth

**Affiliations:** 1 Functional Genetics and Development Division, The Roslin Institute and Royal (Dick) School of Veterinary Studies, University of Edinburgh, Midlothian, EH25 9RG UK; 2 Current Affiliation, Department of Animal Science, Texas A&M University, College Station, Texas, 77843-2471 USA; 3 Clinical Sciences Division, The Roslin Institute and Royal (Dick) School of Veterinary Studies, University of Edinburgh, Midlothian, EH25 9RG UK

**Keywords:** Porcine, Pregnancy, Vitamin D

## Abstract

Emerging evidence suggests an important role of vitamin D in the establishment and maintenance of pregnancy, and the regulation of foetal growth across mammalian species. However, the temporal changes in maternal vitamin D status throughout gestation in the pig and the relationship between maternal vitamin D status and litter characteristics of interest across gestation remain poorly understood and under-investigated. The abundance of 25(OH)D in maternal plasma was quantified by HPLC–MS/MS at gestational days (GD) 18, 30, 45, 60 and 90 (*n* = 5–11 gilts/GD). Maternal plasma 25(OH)D concentrations significantly increased between GD18 and GD30 (*P* < 0.05). The relationship between maternal vitamin D metabolite concentrations and litter characteristics of interest including gilt weight, ovulation rate, mean litter weight, number of live foetuses, percentage prenatal survival, and sex ratio of the litter was assessed. Maternal 25(OH)D (*P* = 0.059) concentrations tended to be positively associated with percentage prenatal survival on GD60. On GD90, maternal 25(OH)D (*P* < 0.05) concentrations were inversely associated with gilt weight. Maternal plasma 25(OH)D concentrations were inversely associated with the percentage of male foetuses in the litter on GD90 (*P* < 0.05). This study has provided novel insights into temporal changes in maternal vitamin D status throughout gestation and the relationship between maternal vitamin D status and the economically important litter characteristics of gilt weight, percentage prenatal survival and percentage of male foetuses in the litter. Improving the understanding of the role of vitamin D across important developmental timepoints in relation to foetal growth is essential to improve reproductive success in livestock species.

**Supplementary Information:**

The online version contains supplementary material available at 10.1186/s40104-022-00760-w.

## Introduction

Postnatally, vitamin D is essential for the maintenance of mineral homeostasis [1] and can be acquired either through the diet or can be synthesized by the skin in response to exposure to ultraviolet irradiation [2]. Nutritional forms of vitamin D consist of plant derived vitamin D_2_ or vitamin D_3_ which is found in oily fish, eggs, and liver. Vitamin D then enters the systemic circulation and is transported to the liver where it is hydroxylated to form the main circulating metabolite of vitamin D (25-hydroxyvitamin D; 25[OH]D). This metabolite is transported to the kidney where it can be further hydroxylated to 1,25-dihydroxyvitamin D (1,25[OH]_2_D) [3]. Binding of vitamin D to the vitamin D receptor (VDR) leads to alteration of the expression of genes and transporters for phosphate and calcium in both the kidney and the intestine, ultimately leading to the maintenance of calcium and phosphate homeostasis [4–6]. Additional postnatal roles of vitamin D include the regulation of cellular proliferation and differentiation, as well as regulation of the immune and nervous systems [7].

In addition to well-characterised roles postnatally, emerging evidence suggests an important role of vitamin D in the establishment and maintenance of pregnancy, and the regulation of foetal growth across mammalian species (reviewed by [8, 9]). Vitamin D insufficiency has been associated with adverse pregnancy outcomes including intrauterine growth restriction [10–12], pre-eclampsia [13, 14], and preterm birth [15–17]. Further, it is known that vitamin D regulatory enzymes and VDR are expressed at the maternal-conceptus interface in many mammalian species, including sheep, humans, rodents, and pigs [18–23], suggesting that local metabolism of vitamin D can occur in the pregnant uterus.

Evidence from multiple species has proven unequivocally that phosphate, calcium, and vitamin D are essential during pregnancy for the appropriate regulation of foetal growth (reviewed by [8, 9]). Yet, the understanding of how maternal vitamin D status changes across gestation, and the association between this and pregnancy outcome and foetal development in the pig remains poorly understood. This study aimed to quantify the maternal circulatory concentration of 25(OH)D across gestation in the pig and to assess whether associations exist between maternal vitamin D status and litter characteristics across gestation. It was hypothesised that maternal plasma 25(OH)D would be positively associated with litter size and mean litter weight throughout gestation.

## Materials and methods

All procedures were performed with approval from The Roslin Institute (University of Edinburgh) Animal Welfare and Ethical Review Board and in accordance with the U.K. Animals (Scientific Procedures) Act, 1986.

### Animal information and sample collection

Large White × Landrace gilts (age 11-14 months) were observed daily for signs of oestrus and were housed indoors in groups of 6-8 animals per pen. Oestrous cyclicity and ovarian function were controlled in accordance with routine normal practice at The Roslin Institute Large Animal Unit [24]. All gilts were inseminated twice daily for the duration of oestrus with semen from one of a four sires (Large White). The first day of insemination was assigned as GD0 and plasma samples were obtained on GD18 (*n* = 5), 30 (*n* = 9), 45 (*n* = 6), 60 (*n* = 12) and 90 (*n* = 9). Gilts were euthanised on the GD of interest with sodium pentobarbitone 20% w/v (Henry Schein Animal Health, Dumfries, Dumfries and Galloway, U.K.) at a dose of 0.4 mL/kg by intravenous injection via a cannula inserted in the ear vein. Immediately before euthanasia, cardiac puncture was performed using an EDTA-coated syringe to collect maternal blood. Plasma was obtained from this sample by centrifugation (1500 × *g* for 15 min at 4 °C) and stored at −20 °C until required.

Following confirmation of death, gilts were hysterectomised. On GD18, the uterine tract was rinsed with saline and string was used to tie the end of the right and left uterine horns at the bifurcation. The uterine horns were cut between the two pieces of string and each uterine horn was placed in a floatation device. The device contained a solution to preserve the integrity of the RNA (700 g ammonium sulphate was dissolved in 935 mL of RNase free water with heat and stirring. Once dissolved, 25 mL of 1 mol/L sodium citrate (Fisher Scientific, Loughborough, Leicestershire, U.K.) and 40 mL of 0.5 mol/L ethylenediaminetetraacetic acid (EDTA) were added. The solution was adjusted to pH 5.2 using concentrated sulphuric acid and stored at room temperature until required. Using dissection scissors, the uterine horn was opened along the mesometrial side, and the conceptuses floated in the solution. Individual conceptuses were removed from the floatation device with forceps and weighed. On GD30, 45, 60, and 90, both uterine horns were dissected from the ovary towards the cervix as described previously [25]. Foetuses were identified as ‘live’ or ‘dead’ based on their morphology at the time of dissection and were weighed. Dead foetuses were excluded from this study. At GD45, 60 and 90, sex was determined by anatomical examination as described previously [26]. DNA was isolated from the GD30 foetuses using the DNeasy Blood and Tissue DNA extraction kit (Qiagen, Manchester, U.K.), and PCR was performed for the sex-determining region Y (Sry) region of the Y chromosome as described previously [24].

The ovaries were dissected to determine the number of corpora lutea present, representing the ovulation rate of the animal. The percentage prenatal survival was then calculated from the ovulation rate, and the number of live conceptuses (embryo/foetus and associated extra-embryonic membranes) present in the uterine tract at the time of euthanasia.

### Determination of 25(OH)D_2_ and 25(OH)D_3_ concentrations in maternal plasma

The concentration of 25(OH)D_2_ and 25(OH)D_3_ metabolites were determined by HPLC–MS/MS analysis as described previously [27].

#### Calibration standards

Eight calibration standards were freshly prepared, by adding 20 µL of 25(OH)D_2_ stock solution (5 µg/mL in ethanol; Sigma-Aldrich, Gillingham, U.K.) and 30 µL of 25(OH)D_3_ stock solution (5 µg/mL in ethanol; Sigma-Aldrich, Gillingham, U.K.) into 1 mL artificial serum [50 mg bovine serum albumin (Sigma-Aldrich, Gillingham, U.K.) were dissolved in 1 mL of phosphate buffered saline], then 1 in 2 serial dilution with artificial serum. The concentrations of calibration standards were 230.8, 115.4, 57.7, 28.9, 14.4, 7.2, 3.6 and 1.8 nmol/L for 25(OH)D_2_; and 356.6, 178.3, 89.2, 44.6, 22.3, 11.2, 5.6 and 2.8 nmol/L for 25(OH)D_3_. These calibration standards were used to generate standard curves for quantification of the concentration of 25(OH)D_2_ and 25(OH)D_3_ in pig plasma by HPLC–MS/MS analysis.

#### Sample preparation

After plasma samples (0.5 mL) were thawed at room temperature, 100 µL of each sample, or calibration standard, was spiked in a 1.5-mL micro-tube with 2 µL of 6,19,19-^2^H_3_-25(OH)D_2_ (1.78 µmol/L; Sigma-Aldrich, Gillingham, U.K.) and 2 µL of 23,24,25,26,27-^13^C_5_-25(OH)D_3_ (2.47 µmol/L; Sigma-Aldrich, Gillingham, U.K.), as internal standards. After adding 20 µL of 1 mol/L NaOH, each plasma sample or calibration standard was then protein precipitated by the addition of 200 µL of acetonitrile. The supernatant of the plasma sample or the calibration standard was purified by solid phase extraction using a Discovery DSC-18 SPE-96 Plate (bed weight: 25 mg/well; Sigma-Aldrich, Gillingham, U.K.). Briefly, the plate was activated with 3 mL of ethyl acetate, 3 mL of methanol and 3 mL of distilled water. After addition of a mixture of supernatant (approximately 300 µL) from protein precipitation and 1 mL 0.4 mol/L K_2_HPO_4_, the plate was washed with 3 mL of distilled water and 2 mL of 40% methanol sequentially and eluted with 1.5 mL of acetonitrile. After evaporating to dryness, samples were derivatized by 2 additions of 25 µL of 0.1 mg/mL DMEQ-TAD (4-[2-(3,4-Dihydro-6,7-dimethoxy-4-methyl-3-oxo-2-quinoxalinyl)ethyl]-3*H*-1,2,4-triazole-3,5(4*H*)-dione; Abcam, Cambridge, U.K.) in ethyl acetate. After evaporation to dryness, derivatized extracts were reconstituted in 25 µL of 60:40 (vol:vol) methanol and 0.1% formic acid:water for HPLC–MS/MS analysis.

#### HPLC–MS/MS analysis

The HPLC–MS/MS analyses were conducted using an UltiMate 3000 HPLC system interfaced to an amaZon ETD tandem mass spectrometer (Bruker Daltonics, Bremen, Germany). Chromatographic separations were achieved using an ACE UltraCore 2.5 SuperC18 column (75 mm × 2.1 mm, 2.5 µm; Advanced Chromatography Technologies, Aberdeen, U.K.), maintained at 40 °C. Gradient elution was performed, with the mobile phase consisting of 10 mmol/L ammonium formate (Fisher Scientific, Loughborough, U.K.) with 0.15% formic acid (buffer A) and methanol with 0.1% formic acid (buffer B). The elution was detected using multiple reaction monitoring with positive electrospray ionisation, with a total runtime of 12 min per sample. Quantitation was carried out using QuantAnalysis 2.0 software (Bruker Daltonics, Bremen, Germany). The standard curve was generated based on the ratio of the peak area of the standard to that of the corresponding internal standard.

### Statistical analyses

All statistical analyses were performed using GenStat 13.1 (VSN International Ltd.) and Minitab. Mean values were calculated for each individual sample for each parameter investigated and the normality of the distribution of the data was assessed by an Anderson–Darling test. If a *P* value of ≤ 0.05 was obtained, then the data were not considered to have a normal distribution. Log10 transformations were carried out where required to achieve a normal data distribution. ANOVA was performed for GD, with a post-hoc Tukey analysis. Linear regressions were performed for total 25(OH)D with ovulation rate, mean litter weight, gilt weight, litter size, and percentage prenatal survival. These regressions were performed both within GD and with all GD combined. Results were considered significant when *P* ≤ 0.05, trending towards significant when 0.05 > *P* < 0.1, and not significant when *P* > 0.1.

## Results

As anticipated, 25(OH)D_2_ was present in small amounts in maternal plasma (2.63 ± 0.19 ng/mL) when compared with 25(OH)D_3_ (58.30 ± 7.41 ng/mL) representing 7.28 ± 0.83% and 92.72 ± 0.83%, respectively, of total 25(OH)D. Total 25(OH)D metabolite concentration is presented in this study and reflects the sum of 25(OH)D_2_ and 25(OH)D_3_ metabolites.

### Temporal changes in maternal vitamin D metabolite abundance

**Fig. 1:**
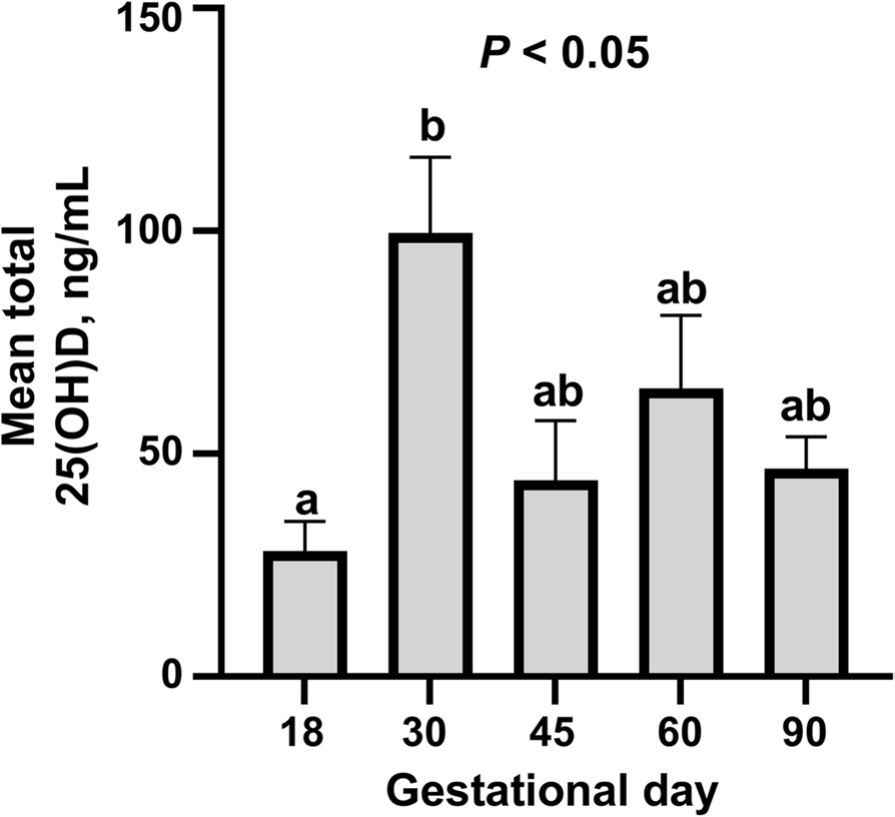
Quantification of maternal total 25(OH)D in maternal plasma on days 18, 30, 45, 60, and 90 of pregnancy. Mean values presented. Error bars represent S.E.M. Different letters indicate that group means differ from one another. *n* = 5–11 gilts per gestational day

Maternal plasma total 25(OH)D (Fig. 1) concentration significantly increased between GD18 and GD30 (*P* < 0.05).

### Associations between maternal circulatory vitamin D metabolite concentrations and litter characteristics

**Fig. 2:**
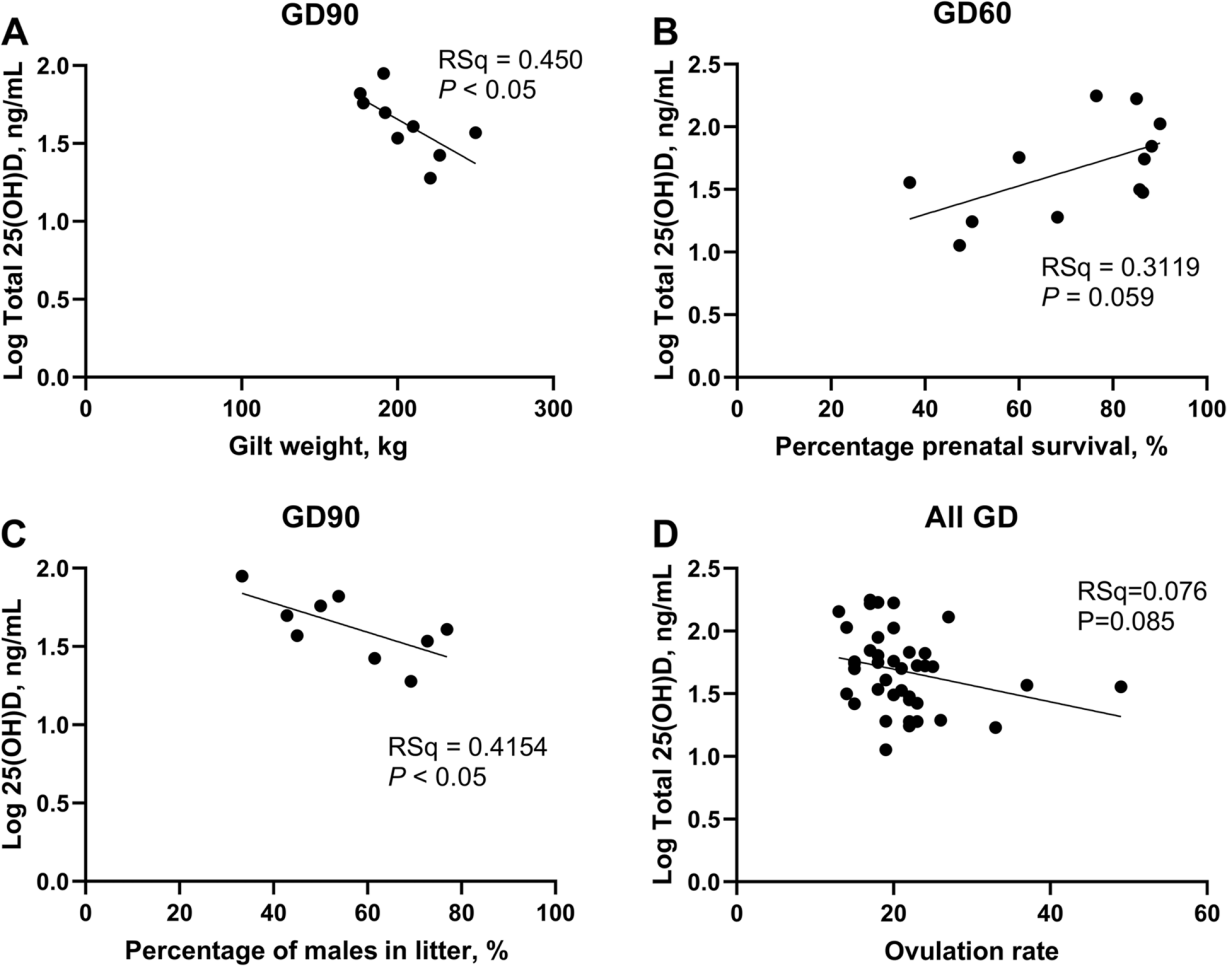
Regressions between gilt weight, percentage prenatal survival, percentage of male foetuses in the litter, and ovulation rate, and maternal total 25(OH)D in maternal plasma on days 18, 30, 45, 60, and 90 of pregnancy. *n* = 5–11 gilts per gestational day

Maternal plasma total 25(OH)D concentrations tended to be positively associated with percentage prenatal survival on GD60 (Table 1 and Fig. 2B; *P* = 0.059). On GD90, maternal plasma total 25(OH)D concentrations were inversely associated with gilt weight (Table 1 and Fig. 2A; *P* < 0.05). An inverse association between maternal plasma total 25(OH)D concentrations and the percentage of male foetuses in the litter was observed on GD90 (Table 1 and Fig. 2C; *P* < 0.05). Maternal plasma total 25(OH)D concentrations tended to be inversely associated with ovulation rate when analysing data with all GD of interest combined (Table 1 and Fig. 2D; *P* = 0.085). Litter size and mean litter weight were not associated with maternal plasma total 25(OH)D concentrations on any GD investigated (Additional File 1: Table S1).

**Table 1:** Regressions between gilt weight, percentage prenatal survival, percentage of males in the litter and ovulation rate, and total 25(OH)D in maternal plasma on days 18, 30, 45, 60, and 90 of pregnancy

Gestational day	Characteristic	Metabolite	RSq	*P*
All	Gilt weight	25(OH)D	0.0153	> 0.10
18	Gilt weight	25(OH)D	0.00068	> 0.10
30	Gilt weight	25(OH)D	0.2700	> 0.10
45	Gilt weight	25(OH)D	0.1297	> 0.10
60	Gilt weight	25(OH)D	0.0086	> 0.10
90	Gilt weight	25(OH)D	0.4504	< 0.05
All	Percentage prenatal survival	25(OH)D	0.06391	> 0.10
30	Percentage prenatal survival	25(OH)D	0.0027	> 0.10
45	Percentage prenatal survival	25(OH)D	0.3360	> 0.10
60	Percentage prenatal survival	25(OH)D	0.3119	0.059
90	Percentage prenatal survival	25(OH)D	0.2774	> 0.10
All	Percentage Mmales in litter	25(OH)D	0.0076	> 0.10
30	Percentage males in litter	25(OH)D	0.2041	> 0.10
45	Percentage males in litter	25(OH)D	0.0170	> 0.10
60	Percentage males in litter	25(OH)D	4.405e-005	> 0.10
90	Percentage males in Llitter	25(OH)D	0.4514	< 0.05
All	Ovulation rate	25(OH)D	0.0757	0.085
18	Ovulation rate	25(OH)D	0.0008	> 0.10
30	Ovulation Rrate	25(OH)D	0.1142	> 0.10
45	Ovulation rate	25(OH)D	0.3951	> 0.10
60	Ovulation rate	25(OH)D	0.029	> 0.10
90	Ovulation rate	25(OH)D	0.0643	> 0.10

## Discussion

While an important role for the transport and metabolism of phosphate, calcium, and vitamin D at the maternal-conceptus interface throughout gestation has been suggested in many species, the function of vitamin D in the regulation of conceptus growth in the pig remains poorly understood. This study provided insights into maternal circulatory vitamin D status across gestation and demonstrated novel interactions between plasma concentrations of vitamin D metabolites and agriculturally important litter characteristics.

While little is known in pigs regarding vitamin D deficiencies during pregnancy it could be speculated that, given the exceptional demands for phosphorous and calcium, this would require alterations in maternal vitamin D homeostasis. Experimentally it has been suggested that piglets born from sows with diets deficient in vitamin D had an increased risk of developing the skeletal abnormality kyphosis [28]. Further, it is well established in production systems where pigs are housed indoors, it is essential to ensure dietary supplementation of vitamin D to prevent the development of hypovitaminosis [29]. Piglets affected by inadequate dietary vitamin D are often lame and reluctant to move, have muscle tremors and hunched back postures, and often die suddenly from hypocalcemia or fractures due to impaired bone development [29]. In contrast, supplementation of sows with vitamin D during pregnancy enhances muscle development of offspring [30]. Improved understanding of the relationship between maternal vitamin D status and conceptus development could improve the understanding of the vitamin D requirements for pregnant sows, which could have significant ramifications for the nutritional management of pregnant sows. It is important to note in this study gilts were utilised as they don’t have the variable metabolic legacies which would be observed using sows, thereby providing an opportunity to investigate the relationship between maternal vitamin D status and litter characteristics in a group of animals that are metabolically uniform. Further studies should elucidate the relationship between maternal vitamin D status in sows but, given the positive impacts of supplementation of vitamin D to sows previously reported, it could be expected that similar associations would be observed in sows.

Maternal plasma total 25(OH)D was greater on GD30 compared to GD18. GD30 corresponds to several important events in pregnancy in the pig [31], including the period of rapid placental growth, and the initiation of bilayer formation and folding. Given the many regulatory roles of vitamin D, it could be speculated that the observed increase in maternal 25(OH)D occurs to increase the availability of vitamin D during this period of high nutritional and metabolic demands. Interestingly, a previous study demonstrated high mRNA expression of vitamin D metabolising enzymes in the ovine placentome and endometria at GD30, accompanied by significant expression of VDR protein by the chorioallantois [23], suggesting that this may not be unique to the pig. Importantly, as the gilts were housed indoors for the duration of the study and the nutritional availability of vitamin D was consistent across gestation, this observed change in maternal vitamin D status may suggest alterations in the maternal regulation of vitamin D metabolism to attempt to increase placental mineral transport during this critical stage of gestation.

Maternal plasma total 25(OH)D concentrations tended to be positively associated with percentage prenatal survival at GD60. It has been suggested that maternal vitamin D status is not associated with the number of lambs born in sheep [27]. However, in that study it was noted that ewes which lost foetuses between the time of pregnancy determination by ultrasound and lambing had lower concentrations of 25(OH)D_2_ and/or 25(OH)D_3_. Additionally, it has been suggested that wild ewes from St. Kilda with higher vitamin D status pre-mating had more lambs surviving to one year of age [32]. Collectively, these findings suggest that in both sheep and pigs there may be a relationship between maternal vitamin D status and foetal, and potentially postnatal, survival which warrants further investigation.

This study demonstrated novel associations between the concentration of 25(OH)D in maternal plasma and the sex ratio of the litter. On GD90, maternal plasma total 25(OH)D was inversely associated with percentage of males in the litter. Across mammalian species it has been demonstrated that sex influences birth weight, with males being heavier than females; a characteristic that can be observed from an early stage of gestation [33–38]. Male porcine conceptuses have been suggested to have an increased growth rate compared to female conceptuses from as early as GD10 [39], which persists throughout gestation [34]. GD90 corresponds to the period of exponential foetal growth in the pig [26], which is associated with increased nutritional, including mineral, demands. Similarly, it has been suggested that the endometrial expression of *VDR* mRNA peaks on day 90 of gestation, and that the endometrial expression of *CYP2R1* (25-hydroxylase) and *CYP27B1* (1-alpha hydroxylase) mRNAs increase with advancing gestational day [22]. Collectively, these findings suggest alterations on both a systemic and a local level in the transport and metabolism of vitamin D in late gestation in pigs. The endochondral skeleton pattern is established early in foetal development however rapid bone formation and mineralization occurs in late gestation. It could be speculated that the inverse association between maternal circulatory 25(OH)D and percentage of males in the litter is reflective of males being larger in late gestation than their female littermates. Therefore, it could be speculated that there would be increased maternal mineral demands to ensure adequate placental mineral transport occurs to meet the extensive nutritional demands of the exponentially growing fetuses. Previous studies in both mice and pigs have demonstrated effects of intrauterine proximity to male foetuses on foetal growth and morphology [40–44]. Additionally, it is known that sex ratio of a litter has consequences for both gilt and boar postnatal reproductive performance [45–52]. Collectively, given the propensity for sex-biased litters and the strong links between sex of other foetuses within a litter and postnatal success, critical interrogation of the association between vitamin D status and conceptus development in sex biased litters may provide important insights to improve pig production systems.

## Conclusion

Emerging evidence suggests a critical role of vitamin D in the establishment and maintenance of pregnancy, and the regulation of conceptus growth across mammalian species. This study suggests in pigs that maternal vitamin D status may be altered from as early as GD30 to ensure appropriate transport of minerals to the conceptus. Further, this has provided novel insights into the relationship between maternal vitamin D status and several economically important litter characteristics across gestation.

## Supplementary Information

**Additional file 1. Table S1: **Regressions between litter size and mean litter weight, and total 25(OH)D in maternal plasma on days 18, 30, 45, 60, and 90 of pregnancy.

## Data Availability

The datasets used and/or analysed during the current study are available from the corresponding author on reasonable request.

## Abbreviations

CYP2R1: 25-Hydroxylase
CYP27B1: 1-Alpha hydroxylase
EDTA: Ethylenediaminetetraacetic acid
GD: Gestational day
HPLC–MS/MS: High performance liquid chromatography-tandem mass spectrometry
PCR: Polymerase chain reaction
Sry: Sex-determining region on the Y chromosome
VDR: Vitamin D receptor
25(OH)D: 25-Hydroxyvitamin D
