# Supplementary material for: Associations between maternal vitamin D status and porcine litter characteristics throughout gestation
Source: J Anim Sci Biotechnol. 2022 Sep 20;13:106. doi: 10.1186/s40104-022-00760-w (PMC9487113; doi:10.1186/s40104-022-00760-w)
Supplement: Supplementary file 1 — Additional file 1. Table S1: Regressions between litter size and mean litter weight, and total 25(OH)D in maternal plasma on days 18, 30, 45, 60, and 90 of pregnancy. [file 40104_2022_760_MOESM1_ESM.docx]

Table S1. Regressions between litter size and mean litter weight, and total 25(OH)D in maternal plasma on days 18, 30, 45, 60, and 90 of pregnancy.

| **Gestational day** | **Characteristic** | **Metabolite** | **RSq** | ***P*** |
| --- | --- | --- | --- | --- |
| All | Litter size | 25(OH)D | 3.797e-005 | >0.10 |
| 30 | Litter size | 25(OH)D | 0.0008 | >0.10 |
| 45 | Litter size | 25(OH)D | 0.0692 | >0.10 |
| 60 | Litter size | 25(OH)D | 0.1214 | >0.10 |
| 90 | Litter size | 25(OH)D | 0.2318 | >0.10 |
| All | Mean litter weight | 25(OH)D | 0.04442 | >0.10 |
| 30 | Mean litter weight | 25(OH)D | 0.1193 | >0.10 |
| 45 | Mean litter weight | 25(OH)D | 0.04212 | >0.10 |
| 60 | Mean litter weight | 25(OH)D | 0.1755 | >0.10 |
| 90 | Mean litter weight | 25(OH)D | 0.04537 | >0.10 |
